# Practical Notes and Formulæ

**Published:** 1882-02-20

**Authors:** 


### PRACTICAL NOTES AND FORMULÆ.

**Preston's Salts** greatly vary in composition. The following is given in Wood and Bache:

| | | |
|---|---|---|
| 1. | Oil of cloves | ½ drachm. |
| | Oil of lavender | 1 drachm. |
| | Oil of bergamot | 2½ drachms. |
| | Strong solution of ammonia | 10 ounces. |

Fill the smelling bottles with coarsely bruised carbonate of ammonia, and add to the salt as much of the aromatic solution as it will absorb. The subjoined gives also very good smelling salts. It is in several respects superior to the above, and is not so generally known as it deserves to be:

| | | |
|---|---|---|
| 2. | Oil of lemon | 1 drachm. |
| | Oil of lavender | ½ drachm. |
| | Oil of cloves | 5 drops. |
| | Stronger ammonia | 15 ounces. |

Fill the smelling bottles with crystallized sulphate of potassa, and pour into each bottle as much of the aromatic ammonia as the salt can retain without spilling. This makes a much prettier looking smelling salt than carbonate of ammonia, and as it does not cake together like it, the bottle need not be emptied when the ammonia has evaporated. All that is necessary is to fill it up again with the aromatic ammonia. The mixture is also more pungent, and its flavor appears to be more generally acceptable to the majority of customers.—[Drug. Circular.

**Anise-seed Soothing Cordial.**—

| | |
|---|---|
| Oil of anise-seed | 3 drachms. |
| Oil of coriander | 5 drops. |
| Deodorized tincture of opium | 17 fluid ounces. |
| Alcohol | 19 pints. |
| Syrup | 20 pints. |
| Water | 40 pints. |
| Cudbear | 2 to 3 drachms. |

Mix together, and after macerating forty-eight hours, filter through paper. Each fluid ounce will contain very nearly half a grain of opium, equivalent to about one-fourth the strength of paregoric. The cudbear is only added, as a precaution, to impart a distinctive color, and may be omitted.—[Druggists' Cir.

**Cathartic Enema.**—

| | | |
|---|---|---|
| ℞ | Senna pulv. } Magnesii sulph. } | aa ℥ j; 30.00 gm. |
| | Aquæ bullentis | Oij; 946.38 fl. gm. |

M. Steep for twenty minutes and strain; then inject the whole gently with the hips raised.—[New Eng. Med. Monthly.

### Formulæ Used in the New York Hospital—For external use

### ECZEMA DRYING SALVE.

℞ Plumbi glycerat........................... ʒ j

Ungt. zinci oxid........................... ℥ j

### KELLY'S TONIC.

℞ Tr. nucis vomicæ.........................f. ʒ ij

Acid. nitromuriat. dil.......................f. ʒ iij

Tr. cinch. co ..............................f. ℥ jss

Tr. gent. co..........................ad f. ℥ iij

Dose, two drachms in water, three times a day.

### HAMILTON'S TONIC.

℞ Strychniæ sulph.......................... gr. viij

Cinchonidiæ sulph........................ ℥ j

Tr. ferri chlor........................... ℥ vj

Syr. zingiberis,

Acid. phosphoric. dil...................aa ℥ xvj

Dose, one teaspoonful three times a day.

### DR. DRAPER.

No. 1.

℞ Acidi citrici,

Ferri et quiniæ cit..................aa ʒ iv

Aquæ,

Syr. limonis........................aa f. ℥ ij

M.

No. 2.

℞ Potass. bicarb. .......................... ʒ iv

Aquæ...................................ad ℥ iv

M One fluid drachm of each in two drachms of water, to be mixed at the time of taking.

### ANTI-RHEUMATIC MIXTURE.

(Mistura Antiarthritica.)

℞ Potassii iodidi........................... ʒv
Vini colchici sem........................ ℥ j
Tr. cimcifugæ rac........................ ℥ ij
Tr. stramon.............................. ℥ ss
Tr. opii camp............................ ℥ jss

M. Dose, ʒ i. three times a day.

### OINTMENT OF IODOFORM.

℞ Iodoform................................ ʒ j

Vaselinæ................................ ℥ j

Reduce the iodoform to powder and add to the vaseline; heat by water bath till dissolved.

COMPOUND IODOFORM OINTMENT.

℞ Pulv. iodoform;

Acidi tannici.......................... aa ʒ j

Vaselinæ ...... ....... ................. ℥ j

OINTMENT OF TAR AND OXIDE OF ZINC.

℞ Ungt. picis... ....... ... .............. ʒ iv

Zinci oxidi .............................. ʒ j

Cerat simpi.............................. ℥ jss

**Aphrodisiacs.**—

According to Dr. Bartholomew, the following are distinctly aphrodisiac combinations in functional generative debility.

℞. Ergot. extract. aquos.......................... ℈j.

Sanguinariæ pulv.............................. gr. ij.

M. For twenty pills. One three times a day.

Or,

℞. Tinct. sanguinar.......... ............ ....... ʒ iij.

Stillingiæ ext., fluid........................... ʒ v.

M. Fifteen to twenty drops in water, thrice daily.—[Druggists' Circular.

**Dewee's Carminative.**—The folllowing is the generally accepted formula for an old and popular preparation:

Carbonate of magnesia.................12 drachms.
Sugar............. .... .......... 3 ounces.
Tincture of assafœtida...... ........... 3 fluid ounces.
Tincture opium......................... 1 fluid ounce.
Water .............................. 24 fluid ounces.

Triturate together until they are mixed.

**Zimmerman's Decoction.**—This is an old preparation, which is directed to be made as follows:

Rhubarb................................. 1 drachm.
Cream of tartar.......................... 1 ounce.
Barley.................................. 1 ounce.
Water................................... 2 pints.

Boil for fifteen minutes, strain, and add enough simple syrup or sugar to sweeten the decoction.

**For Diphtheria.**—Dr. Guttman's prescription:

℞ Pilocarpin muriate.......................... gr. 1½,

Pepsin...................................... ʒss,

Acid muriatic............................. gtts. x,

Aqua ....................................... ℥viij.

M. Teaspoonful every hour.

